# Inflammatory Signaling and Endothelial Activation Drive Thrombosis in Hodgkin and Non-Hodgkin Lymphoma

**DOI:** 10.3390/cells15080667

**Published:** 2026-04-09

**Authors:** Emilija Živković, Olivera Mitrović-Ajtić, Jelena Ivanović, Dragoslava Djikić, Tijana Subotički, Miloš Diklić, Milica Vukotić, Teodora Dragojević, Darko Antić, Vladan P. Čokić

**Affiliations:** 1 Institute for Medical Research, University of Belgrade, Dr Subotica 4, 11129 Belgrade, Serbia; emilija.zivkovic@imi.bg.ac.rs (E.Ž.); tijana@imi.bg.ac.rs (T.S.); milica.tosic@imi.bg.ac.rs (M.V.); teodora.dragojevic@imi.bg.ac.rs (T.D.); 2 Lymphoma Center, Clinic for Hematology, University Clinical Center of Serbia, Dr Koste Todorovića 2, 11000 Belgrade, Serbia; 3 Faculty of Medicine, University of Belgrade, Dr Subotića starijeg 8, 11000 Belgrade, Serbia

**Keywords:** lymphoma, thrombosis, inflammation, nitric oxide, endothelial cells

## Abstract

The high incidence of thrombosis in lymphoma is largely due to chronic inflammation and endothelial dysfunction. To elucidate the mechanisms underlying thrombus formation and fibrinolysis, we investigated interactions between circulating endothelial cells and peripheral blood mononuclear cells (MNCs), along with inflammatory signaling pathways, in patients with follicular lymphoma (FL), Hodgkin lymphoma (HL), and diffuse large B-cell lymphoma (DLBCL), independent of the presence of thrombosis, compared to healthy controls by flow cytometry, immunoblotting, and fluorometric assays. We observed increased tissue factor (TF) expression on CD31+ endothelial cells in DLBCL and FL. In DLBCL, inducible nitric oxide synthase expression was elevated in MNCs, while reduced nitrite levels correlated with an advanced clinical stage in patients with thrombosis. In lymphoma, nuclear factor kappa B (NFκB) signaling was activated in MNCs, while signal transducer and activator of transcription 3 (STAT3) activation was increased in DLBCL with thrombosis. Trans-endothelial migration of MNC was enhanced in HL, FL and DLBCL with thrombosis and reduced by inflammatory cytokine tumor necrosis factor alpha (TNF-α) that promoted platelet aggregation like interleukin-6 (IL-6) in HL and FL. Fibrinolytic analyses showed reduced tissue type plasminogen activator in lymphoma, whereas increased urokinase-type plasminogen activator (uPA) was linked to poorer total survival in DLBCL with thrombosis, suggesting a compensatory role in early thrombus resolution. These findings indicate that chronic inflammation promotes endothelial activation, dysregulated fibrinolysis, and increased vascular permeability, contributing to heightened thrombotic risk. This study provides mechanistic insight into lymphoma-associated thrombosis and identifies TF, uPA, and the inflammatory signaling pathways as potential biomarkers and therapeutic targets.

## 1. Introduction

Thrombotic events represent a major complication in patients with hematological malignancies, particularly lymphomas, and are associated with increased morbidity and mortality [1]. Among common lymphoma subtypes—follicular lymphoma (FL), Hodgkin lymphoma (HL), and diffuse large B-cell lymphoma (DLBCL)—the risk of thrombosis is strongly linked to chronic inflammation [2,3,4]. Cancer-associated inflammation promotes endothelial dysfunction, activation of coagulation pathways, and suppression of fibrinolytic activity, collectively favoring thrombus formation and persistence [5,6,7,8].

Endothelial cells serve as critical regulators in thrombo-inflammatory mechanisms. Upon inflammatory stimulation, endothelial cells and circulating blood cells express tissue factor (TF), the principal initiator of the coagulation cascade [9]. In parallel, inflammation alters the balance between fibrinolytic mediators, including tissue type plasminogen activator (tPA) and urokinase-type plasminogen activator (uPA), thereby impairing clot resolution [10,11]. These alterations establish a prothrombotic vascular milieu that is frequently observed in patients with malignancy.

Several inflammatory signaling pathways implicated in malignancy are also key regulators of thrombosis. Nuclear factor kappa B (NFκB), p38 mitogen-activated protein kinase (MAPK), and Janus kinase–signal transducer and activator of transcription (JAK-STAT) pathways are commonly activated in cancer and amplify thrombo-inflammatory responses through regulation of cytokine production, coagulation factors, and endothelial activation [12,13,14,15]. NFκB and p38 MAPK signaling are frequently upregulated in malignant lymphocytes and have been associated with increased expression of TF and adhesion molecules, further enhancing prothrombotic potential [16,17].

Although HL and non-Hodgkin lymphomas (NHLs), including FL and DLBCL, differ in cellular origin and clinical behavior, they share a common biological feature of persistent immune activation and a cytokine-rich tumor microenvironment. In HL, constitutive activation of NFκB and STAT3 signaling in Reed–Sternberg cells is a hallmark of disease pathogenesis and drives sustained production of pro-inflammatory and procoagulant mediators [18,19]. Similarly, aberrant activation of NFκB, STAT3, and p38 MAPK pathways has been widely documented in NHL, where these pathways regulate tumor cell survival, inflammatory cytokine release, and interactions with the vascular endothelium [20,21]. As such, these signaling cascades represent central mechanistic hubs linking lymphoma-associated inflammation to endothelial dysfunction and thrombotic risk across lymphoma entities.

Endothelial dysfunction is a critical downstream consequence of chronic inflammatory signaling in lymphoma. Nitric oxide (NO) plays a fundamental role in maintaining vascular homeostasis by regulating endothelial tone, platelet activation, and fibrinolysis. Dysregulation of NO synthase (NOS) pathways promotes oxidative stress, platelet reactivity, and impaired fibrinolytic responses, thereby favoring thrombus formation [22]. Despite the recognized clinical importance of thrombosis in lymphoma, the molecular mechanisms driving coagulation activation and impaired fibrinolysis across different lymphoma subtypes, particularly in relation to thrombotic status, remain incompletely understood. In particular, the contribution of endothelial–mononuclear cell (MNC) interactions and inflammatory signaling pathways to the establishment of a prothrombotic milieu in lymphoma patients requires further clarification.

To address these gaps, we investigated the activation of key inflammatory signaling pathways, including NFκB, STAT3, and p38 MAPK, as well as the expression of endothelial NOS (eNOS) and inducible NOS (iNOS) in peripheral blood MNCs, and correlated these findings with clinical parameters in patients with lymphoma. Furthermore, we analyzed the expression of TF and the endothelial adhesion molecule, assessed fibrinolytic regulators and circulating NO levels, and compared these parameters among patients with FL, HL, and DLBCL stratified by thrombotic status. Finally, we evaluated trans-endothelial migration of MNCs and employed a three-dimensional vascular model to investigate the interplay between endothelial cells, platelets, and MNCs under inflammatory conditions. Elucidating these thrombo-inflammatory mechanisms may facilitate the identification of novel biomarkers and therapeutic targets for thrombosis prevention in lymphoma.

## 2. Materials and Methods

### 2.1. The Isolation and Preparation Cells from Peripheral Blood

For the period between 2022 and 2024, the majority of 191 patients with newly diagnosed lymphoma were prospectively enrolled at the Clinic of Hematology, University Clinical Center of Serbia, Belgrade, Serbia. We examined 101 patients with DLBCL involving 43 females (age 57.56 ± 16.29 years) and 58 males (age 57.98 ± 13.71 years), 46 patients with FL involving 35 females (age 57.69 ± 11.46 years) and 11 males (age 59.82 ± 11.71 years), and 44 patients with HL involving both 22 females (age 36.32 ± 10.6 years) and males (age 29.68 ± 13.26 years). We also examined 17 healthy controls involving 10 females (age 38.7 ± 9.87 years) and 7 males (age 41.14 ± 9.15 years). Laboratory and clinical data of patients with lymphoma were collected at diagnosis. Thrombosis was determined in accordance with observed acute myocardial infarction, stroke, venous and arterial thrombosis, deep vein thrombosis, superficial thrombosis, and pulmonary embolism before, during or after chemotherapy. Informed consent was obtained from all of the participants included in the study and approved by the local Ethical Committee. Peripheral blood (30 mL) from DLBCL, FL and HL patients was used to isolate MNCs, endothelial cells, neutrophils, and platelets. Collected blood (EDTA, BD Vacutainer K2A, Eysins, Switzerland) was processed by gradient centrifugation using Lymphocyte Cell Separation Media (Capricorn Scientific, Ebsdorfergrund, Germany). Circulating endothelial cells were isolated from whole blood using BD FACS lysing solution (BD Bioscience, 349202, Franklin Lakes, NJ, USA). Proteins were isolated from whole cell pellets of MNCs and used for Western blot analysis. CD markers were quantified by flow cytometry in MNCs, endothelial cells, neutrophils, and platelets.

### 2.2. Flow Cytometry

Cell surface expression of CD markers was analyzed by flow cytometry. Mononuclear cells (MNCs), neutrophils, platelets, endothelial cells, and platelet–monocyte aggregates were fixed in 4% paraformaldehyde and stained with antibodies conjugated to fluorescein isothiocyanate (FITC), phycoerythrin (PE), allophycocyanin (APC), or peridinin chlorophyll protein complex/cyanine5.5 (PerCP/Cy5.5). The following antibodies were used: CD142-APC (BioLegend, 365206, San Diego, CA, USA), CD31-PE (BioLegend, 303106), CD144-PE (BioLegend, 348506), and CD146-PerCP/Cy5.5 (BioLegend, 361010). Labeled cells were analyzed on a BD FACSCalibur (BD Biosciences, Franklin Lakes, NJ, USA), with a minimum of 10,000 events recorded per sample. Data analysis was performed using FlowJo v10.8.1 software (FlowJo LLC, Ashland, OR, USA).

### 2.3. Tissue Type Plasminogen Activator and Urokinase Activity

The fibrinolytic capacity of patient plasma was assessed by measuring the activity of two plasminogen activators. Tissue type plasminogen activator (tPA) activity was determined using a Tissue Type Plasminogen Activator Activity Assay Kit (Abcam, ab108905, Waltham, MA, USA), following the manufacturer’s protocol. This assay evaluates the ability of tPA present in human plasma to convert plasminogen into plasmin. The generated plasmin cleaves a specific chromogenic substrate, releasing para-nitroaniline (pNA), which produces a yellow color. The increase in absorbance of pNA was measured spectrophotometrically at 405 nm. Urokinase activity was quantified using a Urokinase Activity Fluorometric Assay Kit (Sigma-Aldrich, Burlington, MA, USA). In this assay, enzymatic cleavage of the provided substrate produces a fluorescent signal, with excitation at 350 nm and emission at 450 nm. The intensity of fluorescence is directly proportional to the urokinase activity present in the sample.

### 2.4. Cell Culture

Human microvascular endothelial cell-1 (HMEC-1) is an endothelial-like cell isolated from the endothelium of the foreskin of a male patient. HMEC-1 cells were supplied by ATCC (TIB-180, Manassas, VA, USA). Cells were cultured in a microvascular endothelial cell growth medium kit enhanced (PELOBiotech, Planegg, Germany) supplemented with 10% FBS, 1% glutamine, and 1% penicillin/streptomycin. Cells were incubated at 37 °C and 5% CO_2_ and maintained between 7 × 10^5^ and 1 × 10^6^ cells/mL, rinsed with PBS, and fresh medium was added every 2 days. In all experiments, cells were treated with inflammatory cytokine tumor necrosis factor alpha (TNF-α, 5 ng/mL) and interleukin-6 (IL-6, 20 ng/mL, Miltenyi Biotec, Bergisch Gladbach, Germany). HMEC-1 (5 × 10^5^ cells/well) were seeded on a 4 μm pore membrane, in the upper compartment of Boyden chamber. Also, 30 µL of 1 × 10^6^ cells/mL suspension was seeded in microchip flow chamber.

### 2.5. Western Blot

Whole cell extract was obtained from cell pellets of MNC by extraction in RIPA buffer (1 mM EDTA, 50 mM Tris-HCl pH 7.5, 0.1% SDS, 150 mM NaCl, 1% NP40, 1% sodium deoxycholate, protease inhibitor cocktail). Homogenates were separated into polyacrylamide gels and blotted to polyvinylidene difluoride membranes (GE Healthcare, Chicago, IL, USA). Signal has been detected using antibodies directed against phospho-NFκB (Ser536) (3031, Cell Signaling, Danvers, MA, USA), total NFκB (E-AB-22066, Elabscience, Houston, TX, USA), phospho-p38 (Tyr180/Tyr182) (E-AB-21027, Elabscience), total p38 (E-AB-66279, Elabscience), STAT3 (Cell Signaling, 9132, Danvers, MA, USA) and pSTAT3 Tyr705 (Cell Signaling, 9131S) and incubated overnight at 4 °C. Secondary antibodies conjugated to horseradish peroxidase (Elabscience) were detected using an enhanced chemiluminescence detection system (BioRad Laboratories, Hercules, CA, USA). Protein bands were visualized using ChemiDoc Imager (BioRad Laboratories) and quantified in ImageLab software (BioRad Laboratories, Version 6.0.0.25).

### 2.6. RT-qPCR

Relative expressions of endothelial nitric oxide synthase (eNOS) and inducible nitric oxide synthase (iNOS) transcripts were analyzed by quantitative reverse transcription polymerase chain reaction (RT-qPCR). Granulocyte pellets collected from lymphoma patients and healthy volunteers were used for RNA extraction. Total RNA was isolated using Trisure™ reagent (Bioline Ltd., London, UK) in accordance with the manufacturer’s guidelines. RNA quantity and quality were evaluated spectrophotometrically using a Nanophotometer P330 (Implen GmbH, Munich, Germany). For cDNA synthesis, equal amounts of purified RNA from each sample were reverse-transcribed using the High-Capacity cDNA Reverse Transcription Kit with RNase Inhibitor (Thermo Fisher Scientific, Waltham, MA, USA), following the supplier’s instructions. Quantitative PCR reactions were then carried out with the FastGene 2× IC Green Universal Kit (Nippon Genetics, Düren, Germany) using a Magnetic Induction Cycler system (Bio Molecular Systems, Upper Coomera, QLD, Australia). Each sample was analyzed in at least five technical replicates to ensure reproducibility of the measurements. Gene expression levels were normalized to the β-actin housekeeping gene. Relative transcript levels were calculated using the comparative Ct (2^−ΔΔCt^) approach with Magnetic Induction Cycler Software version 2.10.5 (Bio Molecular Systems Pty Ltd., Upper Coomera, QLD, Australia). The following primer pairs were used for amplification: eNOS forward 5′-CGG CAT CAC CAG GAA GAA GA-3′ and reverse 5′-GCC ATC ACC GTG CCC AT-3′ (Invitrogen, Thermo Fisher Scientific, Carlsbad, CA, USA); iNOS forward 5′-CCA GCC CTC AGA GTA CAG-3′ and reverse 5′-GGG ACC CTG GCC ATC T-3′ (Integrated DNA Technologies, Coralville, IA, USA); and β-actin forward 5′-CCT GGC ACC CAG CAC AAT-3′ with reverse 5′-GCC GAT CCA CAC GGA GTA CT-3′ (Invitrogen, Thermo Fisher Scientific, Carlsbad, CA, USA).

### 2.7. Boyden Chamber

To follow the migration of MNCs in patients and healthy donors across the endothelial barrier to the lower chamber, a 24-well cell migration assay was used (Cell Biolabs, Inc., CBA-102, San Diego, CA, USA). HMEC-1 cells were seeded monolayer on 4 μm pore membrane in the Boyden chamber’s upper compartment with or without TNF-α treatment (5 ng/mL). After 24 h, MNC (2 × 10^5^ cells/well) was added, co-cultured with a monolayer of HMEC-1 and incubated for 24 h. The migratory cells were labeled with CyQuant GR dye and fluorescence measurement (×100,000) was performed with the Perkin Elmer Wallac 1420 Victor2 Microplate Reader (Turku, Finland) at 480/520 nm.

### 2.8. Automated Microchip Flow Chamber System

Quantitative assessment of thrombus formation during flow conditions was performed using an automated microchip flow chamber platform. HMEC-1 cells (30 µL of 1 × 10^6^ cells/mL suspension) with or without treatment of TNF-α (5 ng/mL) and IL-6 (20 ng/mL) were seeded on 15 µ-Slide VI (Ibidi, 80606, Gräfelfing, Germany) which were pre-coated with 100 µL of 15 µg/mL collagen type I and nuclei were labeled with DAPI stain (Sigma, D-9542). Suspensions of platelets and MNCs of patients and healthy donors (ratio 50:1–PLR ratio, resuspended in RPMI-1640 (BioWest, Lakewood Ranch, FL, USA) were passed through the flow using an Aladdin SyringeTWO Programmable Double Syringe Pump (WPI, AL-4000, Sarasota, FL, USA). MNCs were pre-stained with CFSE dye, while platelets were labeled with PerCep-cy5.5 conjugated CD41a (BioLegend, 303720, San Diego, CA, USA). Imaging was performed using an epifluorescence microscope (Olympus Provis AX70, Tokyo, Japan). Quantification of fluorescence area that platelets occupy and number of platelets was measured in Fiji (Image J, version 1.53) (National Institutes of Health (NIH), Bethesda, MD, USA). Percentage of positive cells was calculated based on total image area.

### 2.9. Ozone-Based Chemiluminescent Determination of Nitrite in Human Plasma

For nitrite (NO^2−^) measurements, plasma samples from patients and healthy donors were deproteinized by adding cold 96% ethanol (500 µL plasma to 1 mL ethanol), followed by shaking for 30 min at 0 °C. The samples were then centrifuged at 14,000 rpm for 10 min at 4 °C, and the supernatants were transferred to fresh tubes. Nitrite levels were determined using the Sievers Model 280 Nitric Oxide Analyzer (Sievers, Boulder, CO, USA) as previously described [23]. Nitrite was chemically reduced to NO using an acidified potassium iodide (KI) solution composed of 4 mL glacial acetic acid, 1 mL distilled water, and 50 mg KI.

### 2.10. Statistical Analysis

The distribution of the data was evaluated using the Shapiro–Wilk and Kolmogorov–Smirnov normality tests. Group comparisons were performed using Student’s *t*-test implemented in GraphPad Prism software (version 8.0.0 for Windows; GraphPad Software Inc., San Diego, CA, USA). In cases where the data did not follow a normal distribution, the nonparametric Mann–Whitney test was applied for comparisons between groups. A *p*-value below 0.05 was considered to indicate statistical significance.

## 3. Results

### 3.1. Adhesion Molecule and Tissue Factor Expression on Circulating Endothelial Cells in Lymphoma

**Figure 1:**
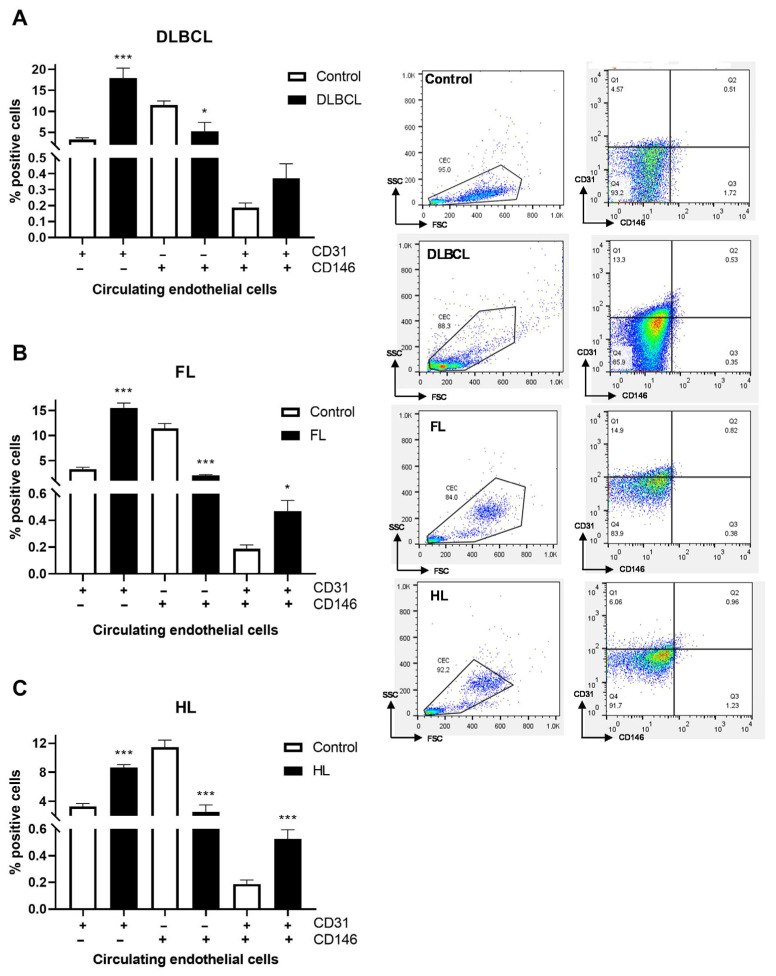
Adhesion molecule CD146 expression in circulating endothelial cells in lymphoma patients. Graphs represent the expression of CD31 and CD146 on circulating endothelial cells in (**A**) diffuse large B-cell lymphoma (DLBCL), (**B**) follicular lymphoma (FL) and (**C**) Hodgkin lymphoma (HL) compared to healthy controls (control, *n* = 4). Next to the graphs are gating images for CD31 and CD146 markers’ distribution by flow cytometry. Values are mean ± SEM (*n* = 6–7 for lymphomas). * *p* < 0.05, *** *p* < 0.001 vs. corresponding control.

**Figure 2:**
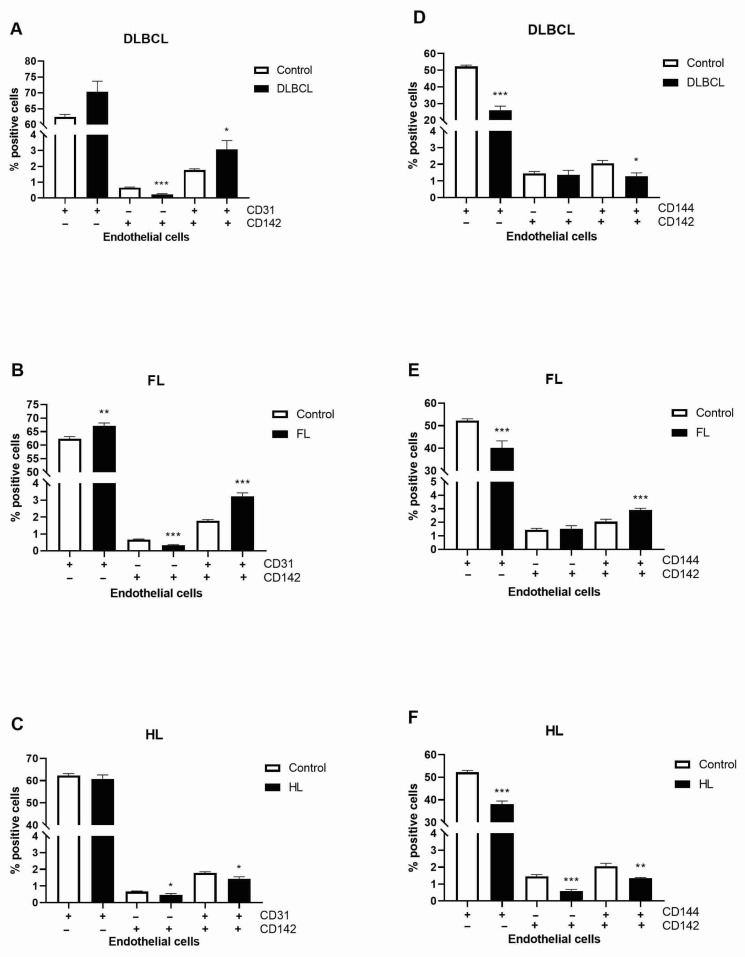
Tissue factor expression in circulating endothelial cells in lymphoma patients. Flow cytometry was used to assess levels of endothelial CD31 marker and tissue factor (CD142) in (**A**) diffuse large B-cell lymphoma (DLBCL), (**B**) follicular lymphoma (FL) and (**C**) Hodgkin lymphoma (HL) in comparison to healthy volunteers (control). Graphs also represent expression of endothelial CD144 marker and CD142 positive cells in (**D**) DLBCL, (**E**) HL and (**F**) FL in comparison to control. The gating images for CD31, CD142 and CD144 markers’ distribution by flow cytometry are presented in Supplemental Figure S1. Values are mean ± SEM (*n* = 5). * *p* < 0.05, ** *p* < 0.01, *** *p* < 0.001 vs. corresponding control.

We examined the adhesive and activation properties of lymphoma-derived CD31+ endothelial cells by analyzing the expression of CD146, a multifunctional endothelial molecule mediating leukocyte adhesion, trans-endothelial migration, and vascular dysfunction [24]. Importantly, the frequency of CD31+ CD146+ events detected in our samples was very low, which is consistent with the expected rarity of circulating endothelial cells and reduces the likelihood of significant contamination by other cell types. The circulating CD31+ CD146+ cells were significantly elevated on FL and HL endothelial cells, with a similar trend observed in DLBCL (Figure 1A–C). To directly assess the thrombogenic potential of circulating endothelial subsets, we analyzed the expression of TF (CD142) on CD31+ and CD144+ endothelial cells, as TF represents the principal initiator of coagulation. Increased TF expression on endothelial cells is a well-established indicator of endothelial procoagulant activation and thrombotic potential [25]. By comparing TF expression between CD31+ endothelial cells, which are primarily associated with leukocyte transmigration and endothelial activation, and CD144+ endothelial cells, which reflect endothelial junctional integrity and barrier function [26,27], we aimed to distinguish endothelial phenotypes linked to prothrombotic versus barrier-preserving functions. Notably, circulating CD31+ endothelial cells were increased, whereas CD144+ cells were decreased across lymphoma subtypes compared to healthy donors (Figure 2A–C, Supplemental Figure S1). TF expression was increased on CD31+ endothelial cells in patients with DLBCL (*p* < 0.05) and FL (*p* < 0.001) but reduced in HL compared to healthy controls (*p* < 0.05; Figure 2A–C). In contrast, TF expression on CD144+ endothelial cells was elevated in FL (*p* < 0.001) but decreased in DLBCL (*p* < 0.05) and HL (*p* < 0.01) relative to controls (Figure 2D–F). CD31+ endothelial cells are characterized by increased CD146 and TF expression, indicating increased adhesive and procoagulant properties of malignant endothelial cells in non-Hodgkin lymphoma (NHL).

### 3.2. Impaired Fibrinolysis and Increased Leukocyte/Monocyte Migration in Lymphoma Patients

**Figure 3:**
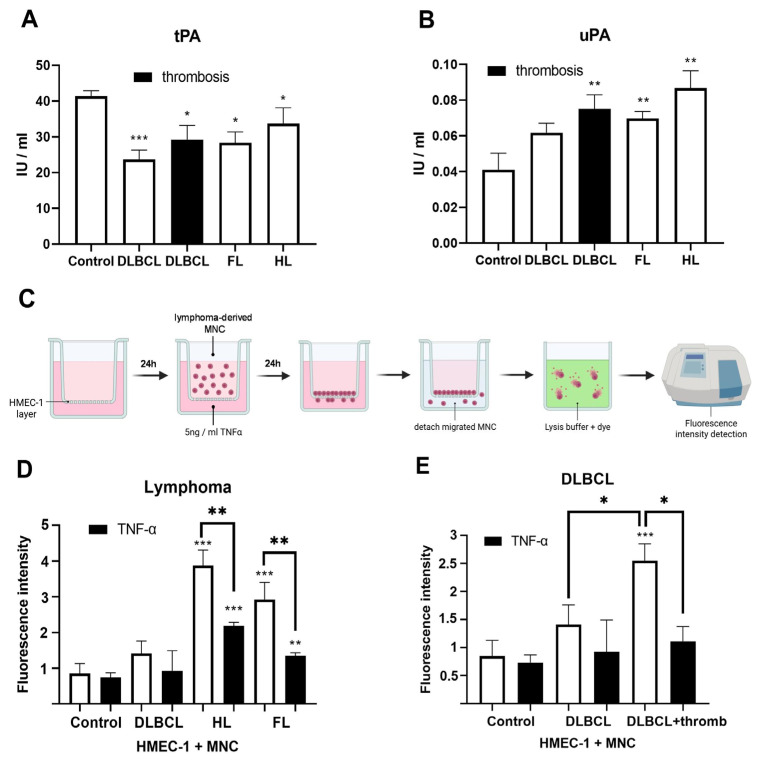
Fibrinolytic biomarker profiles in lymphomas. Fibrinolysis factor quantification: (**A**) tissue plasminogen activator (tPA) and (**B**) urokinase plasminogen activator (uPA) in plasma of peripheral blood of healthy volunteers (control, *n* = 5) and patients of follicular lymphoma (FL, *n* = 24–27), Hodgkin lymphoma (HL, *n* = 18), and diffuse large B-cell lymphoma (DLBCL) with (*n* = 16) or without (*n* = 20–22) thrombosis. (**C**) Schematic representation of the trans-endothelial migration assay: human microvascular endothelial cells (HMEC-1) were cultured on the transwell insert and stimulated with 5 ng/mL tumor necrosis factor alpha (TNFα) for 24 h. Lymphoma-derived mononuclear cells (MNCs) were added to the upper chamber and allowed to migrate for 24 h. (**D**) Fluorescence intensity indicating migration of MNCs from DLBCL (*n* = 5), HL (*n* = 4), and FL (*n* = 3) and healthy controls (*n* = 4) treated with TNFα or vehicle (non-treated). Open boxes are controls, while black boxes are TNF-α treated. (**E**) Fluorescence intensity indicating migration of MNCs from DLBCL patients with (*n* = 4) or without thrombosis (*n* = 5) treated with TNFα or vehicle (non-treated). Migrated MNCs were detached, lysed, and fluorescence intensity was measured. Values are mean ± SEM. * *p* < 0.05, ** *p* < 0.01, *** *p* < 0.001 vs. control.

Plasminogen conversion to plasmin, a key step in fibrinolysis, is mediated by uPA and tPA released by endothelial cells [28,29]. The tPA was reduced in the plasma of lymphoma patients, particularly in DLBCL patients without thrombosis (*p* < 0.001; Figure 3A). In contrast, uPA levels were generally elevated in lymphoma patients and were significantly higher in DLBCL patients with thrombosis compared to healthy controls (*p* < 0.01; Figure 3B). The uPA levels were in positive correlation (Spearman) with β2 mikroglobulin (β2M, ρ = 0.557, 95% CI = 0.046–0.837, *p* = 0.034) in HL. The tPA level was in negative correlation with MCHC (Pearson, ρ = −0.43, r^2^ = 0.185, 95% CI = −0.715–−0.022, *p* = 0.041) in FL. The uPA levels were in negative correlation (Spearman) with overall survival (ρ = −0.601, 95% CI = −0.802–−0.275, *p* = 0.001) in FL. Increased endothelial permeability can occur in chronic inflammation and cancer [30,31]. We assessed the migration of lymphoma-derived MNCs through HMEC-1 using a Boyden chamber (Figure 3C). Trans-endothelial leukocyte/monocyte migration was elevated in patients with HL and FL compared to healthy volunteers (*p* < 0.001), while TNF-α reduced their migration (*p* < 0.01; Figure 3D). In DLBCL patients with thrombosis, MNC migration was higher than in healthy volunteers (*p* < 0.001) and DLBCL patients without thrombosis (*p* < 0.05), while TNF-α similarly reduced migration (*p* < 0.05; Figure 3E). Together, decreased tPA indicates impaired fibrinolysis, while increased uPA and trans-endothelial migration of MNCs suggest increased endothelial permeability in lymphoma compared to healthy controls.

### 3.3. Activation of Inflammatory Signaling Pathways in Lymphoma

**Figure 4:**
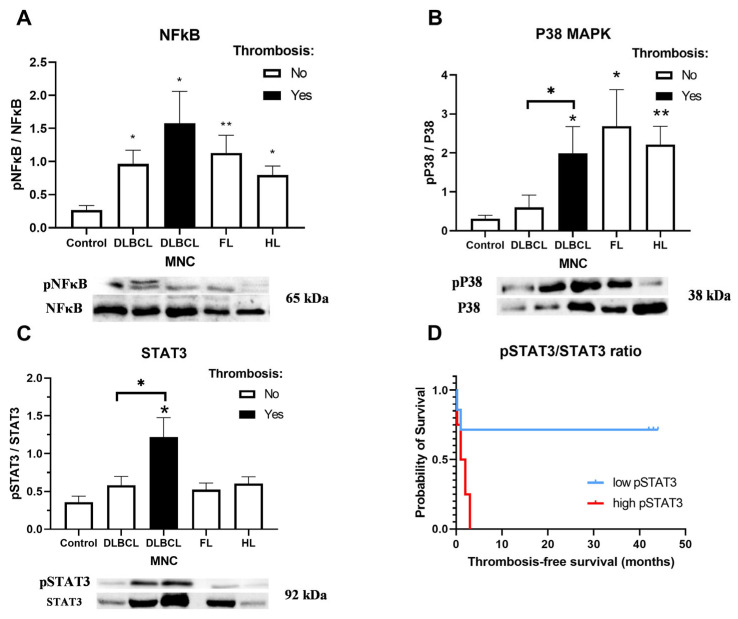
Activation of inflammatory signaling pathways in lymphomas. Peripheral blood mononuclear cells (MNCs) were isolated from patients with diffuse large B-cell lymphoma (DLBCL) with (*n* = 15–19) or without thrombosis (*n* = 9–11), follicular lymphoma (FL, *n* = 9–11) and Hodgkin lymphoma (HL, *n* = 10–12) and activation of (**A**) NFκB, (**B**) STAT3 and (**C**) p38 MAPK signaling pathways were analyzed relative to those in healthy volunteers (control, *n* = 10). (**D**) Kaplan–Meier curve presenting thrombosis-free survival in DLBCL patients stratified by pSTAT3/STAT3 ratio in MNC. Tick marks indicate censored observations. Representative immunoblot images corresponding to the graphs, showing phosphorylated signaling proteins and their respective total protein levels (used for ratio quantification). Values are mean ± SEM. * *p* < 0.05, ** *p* < 0.01 vs. control or as indicated.

Inflammatory disorders, such as clonal hematopoiesis of indeterminate potential, are associated with an increased risk of thrombosis [32]. We analyzed inflammatory NFκB, STAT3, and p38 MAPK signaling pathways in MNCs, fractions of which include lymphocytes and monocytes involved in thrombus resolution [32]. NFκB activation was elevated in lymphoma patients compared to healthy controls, with the highest levels observed in DLBCL patients with thrombosis (Figure 4A). STAT3 activation was specifically increased in DLBCL with thrombosis relative to both healthy volunteers and DLBCL without thrombosis (*p* < 0.05; Figure 4B). p38 MAPK signaling was generally elevated in lymphomas compared to healthy controls and was also significantly higher in DLBCL with thrombosis compared to both healthy volunteers and DLBCL without thrombosis (*p* < 0.05; Figure 4C). Table 1 demonstrates correlation analysis between activation of inflammatory signaling pathways and clinical and laboratory parameters in patients with different lymphoma subtypes (DLBCL, HL, and FL). All available clinical and laboratory variables recorded for the patient cohorts were included in an exploratory correlation analysis. For clarity and conciseness, only statistically significant correlations are presented in the table. Consequently, the parameters shown differ between lymphoma subtypes and reflect variables for which significant associations with pathway activation were identified, rather than a predefined set of clinical comparisons. Activation of STAT3 signaling was in negative correlation with the Khorana risk score for venous thromboembolism in DLBCL and HL as well as DLBCL stage (Table 1). Activation of NFκB signaling was in positive correlation with INR and an absolute neutrophil count in HL (ANC, Table 1). Kaplan–Meier analysis demonstrated significantly shorter thrombosis-free duration in the high pSTAT3/STAT3 level group compared with low pSTAT3/STAT3 (Figure 4D, *p* = 0.04). Median thrombosis-free survival was 1.5 months in the high pSTAT3/STAT3 group (95% CI = 0.025–0.955) and not reached for 36 months in the low pSTAT3/STAT3 group. However, ROC analysis indicated that the predictive accuracy of the biomarker was not statistically significant (Supplemental Figure S2A), with an AUC of 0.73 (95% CI = 0.4–1; *p* = 0.2), likely due to a limited sample size warranting validation in a larger, independent cohort. Reduced mobility was 3.6-fold more present in DLBCL patients with thrombosis (Mann–Whitney test, *p* = 0.0375) compared to the patients without thrombosis. Smoking status was similar between DLBCL patients with and without thrombosis: 7/22 (31.8%) and 25/79 (31.6%) were smokers, respectively, while smoking status was unknown in three and nine patients. NFκB and p38 inflammatory signaling pathways were generally activated in lymphoma, while STAT3 is enhanced specifically in thrombotic DLBCL patients.

### 3.4. Nitric Oxide Synthase Activity in Vasculature of Lymphomas

**Figure 5:**
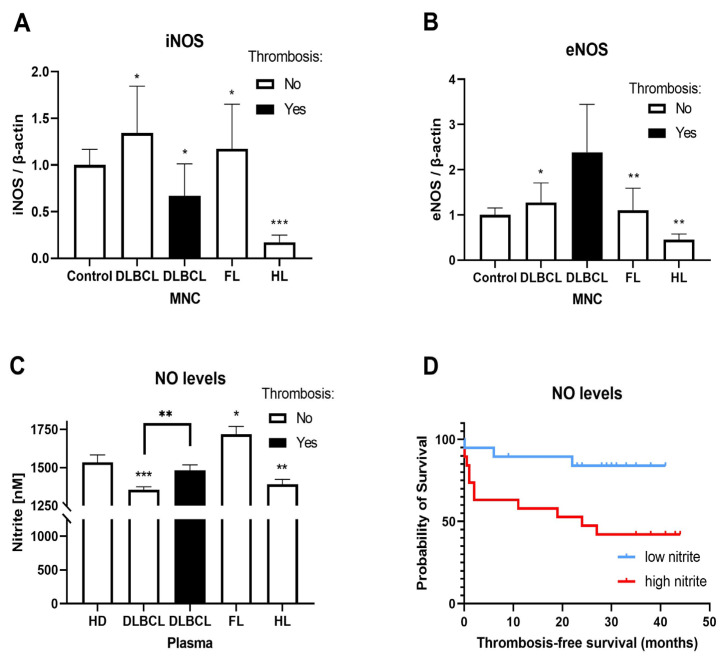
Nitric oxide synthase expression and activity in mononuclear cells of lymphoma. The levels of (**A**) endothelial (eNOS) and (**B**) inducible (iNOS) nitric oxide synthase gene expression in mononuclear cells (MNCs) of DLBCL (*n* = 23), HL (*n* = 7), FL (*n* = 14) and control (*n* = 6). (**C**) Nitrite levels in plasma of peripheral blood of patients with DLBCL with (*n* = 20) or without thrombosis (*n* = 25), HL (n = 19), FL (*n* = 17) and control (*n* = 6). (**D**) Kaplan–Meier curve presenting thrombosis-free survival in DLBCL patients stratified by plasma nitrite levels. Tick marks indicate censored observations. Values are mean ± SEM. * *p* < 0.05, ** *p* < 0.01, *** *p* < 0.001 vs. corresponding control or as indicated.

NOS inhibitors have anti-inflammatory effects, whereas NO can increase vascular permeability and cell migration [33]. We examined eNOS and iNOS mRNA expression in MNCs and plasma nitrite levels in the plasma of lymphoma patients compared to healthy donors. We observed increased iNOS gene expression in patients with FL and DLBCL without thrombosis, but reduced expression in HL and DLBCL with thrombosis compared to healthy donors (Figure 5A). In addition, eNOS gene expression was also increased in patients with FL and DLBCL without thrombosis, but with reduced expression in HL (Figure 5B). Furthermore, iNOS expression was in positive correlation (Spearman) with eNOS expression in DLBCL (ρ = 0.801, 95% CI = 0.572–0.914, *p* < 0.0001) and HL (ρ = 0.801, *p* = 0.034), while in negative correlation with aPTT (ρ = −0.618, 95% CI = −0.893–0.009, *p* = 0.048) and albumin (ρ = −0.647, 95% CI = −0.902–0.056, *p* = 0.036) in FL. Moreover, eNOS expression was in positive correlation (Pearson) with MCHC (ρ = 0.827, r^2^ = 0.684, 95% CI = 0.046–0.98, *p* = 0.042) and in negative correlation with RDW (ρ = −0.827, r^2^ = 0.684, 95% CI = −0.98–048, *p* = 0.042) in HL. Plasma NO levels (metabolite nitrite) were elevated in FL patients but reduced in patients with DLBCL or HL (Figure 5C). The presence of thrombosis restored nitrite levels to values comparable to those of healthy donors (Figure 5C). Kaplan–Meier analysis of thrombosis-free survival according to plasma nitrite levels revealed a significant difference between high and low nitrite groups (Figure 5D, *p* = 0.01). The median thrombosis-free survival was 24 months in patients with high NO groups, meaning that in 2 years 50% of those patients had developed thrombosis (Figure 5D). However, the median was undefined in the low NO subset since less than 50% of patients experienced thrombosis within 44 months of follow-up (Figure 5D). Plasma nitrite levels independently demonstrated significant value as thrombosis predictor with AUC of 0.71 (*p* = 0.032, Supplemental Figure S2B). While Khorana score in our cohort did not show significant predictive power (AUC 0.62, *p* = 0.21, Supplemental Figure S2F), the addition of plasma nitrite levels improved AUC to 0.71 in a combined model (*p* = 0.02, Supplemental Figure S2D). Furthermore, a model combining ThroLy score and plasma nitrite levels showed a superior predictive performance with an AUC of 0.79 (95% CI = 0.64–0.95; *p* = 0.0027, Supplemental Figure S2C), compared to both predictors independently (AUCs of 0.73, *p* = 0.019 and 0.71, *p* = 0.032, respectively, Supplemental Figure S2B,E). NO levels were in negative correlation with clinical stage (ρ = −0.534, 95% CI = −0.795–−0.106, *p* = 0.0154) in DLBCL with thrombosis. NO levels were in positive correlation with fibrinogen (ρ = 0.44, 95% CI = 0.042–0.72, *p* = 0.028), CRP (ρ = 0.522, 95% CI = 0.147–0.765, *p* = 0.008) and platelet-to-lymphocyte ratio (PLR, ρ = 0.421, r^2^ = 0.177, 95% CI = 0.031–0.7, *p* = 0.036) in DLBCL. Moreover, NO levels were in positive correlation with INR (ρ = 0.561, 95% CI = 0.095–0.826, *p* = 0.021) and PTs (ρ = 0.578, 95% CI = 0.119–0.833, *p* = 0.017) in FL. We demonstrated increased NO production in the vasculature of FL and decreased NO production in DLBCL and HL, where the former was restored after thrombus formation.

### 3.5. Inflammation Induced Endothelium Permeability and Thrombus Formation in Lymphomas

**Figure 6:**
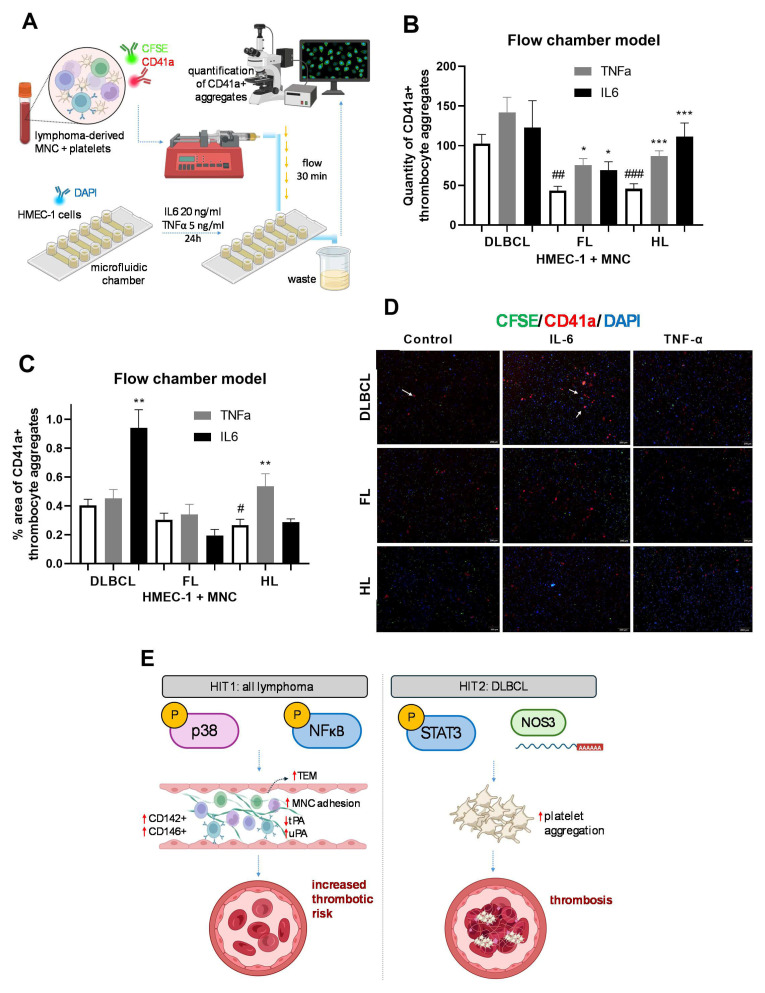
Inflammatory stimulation of platelets aggregation in lymphoma. (**A**) Schematic representation of the experimental setup for the co-culture of HMEC-1 endothelial cells, lymphoma-derived mononuclear cells (MNCs), and platelets during 24 h of incubation using an automated microfluidic flow chamber system. HMEC-1 cells were pretreated with tumor necrosis factor alpha (TNFα, 5 ng/mL) and interleukin-6 (IL-6, 20 ng/mL) for 24 h before the introduction of labeled MNCs and platelets. Open boxes represent control samples, gray boxes indicate TNF-α–treated samples, and black boxes correspond to IL-6–treated samples. After 30 min of flow, immunofluorescence staining was performed, and the number and area of CD41a+ platelet aggregates were quantified. (**B**) Quantity and (**C**) area of CD41a+ platelet aggregates from DLBCL (*n* = 4), FL (*n* = 3) and HL (*n* = 5) patients co-cultured with HMEC-1 cells pretreated with TNFα, IL-6 or vehicle. (**D**) Immunofluorescence images show platelet aggregates stained with CD41a antibody (white arrows), MNC labeled with CFSE (green), and HMEC-1 nuclei stained with DAPI (blue) in the microfluidic flow chamber. (**E**) Schematic diagram represents our two-hit model of inflammation-driven thrombosis development in lymphoma: NFκB and p38 activation drives endothelial activation across all lymphoma subtypes, leading to an increased thrombotic risk. The critical molecular event for thrombosis occurrence in DLBCL is IL6/STAT3-driven platelet aggregation combined with loss of NO-mediated vascular protection. Values are mean ± SEM. * *p* < 0.05, ** *p* < 0.01, *** *p* < 0.001 vs. corresponding non-treated lymphomas (*) and # *p* < 0.05, ## *p* < 0.01, ### *p* < 0.001 vs. DLBCL (#). Abbreviations: TEM, transendothelial migration.

To observe endothelial adhesion, we generated a 3D model of a blood vessel using a microfluidic chip on which we seeded HMEC-1, treated with TNF-α or IL6, and passed pre-stained lymphoma-derived MNC and platelets under blood flow conditions (Figure 6A). Single-stain controls for endothelial HMEC-1 cells, mononuclear cells, and platelets are shown in Supplemental Figure S3, confirming the integrity of the endothelial layer under the applied flow conditions. DLBCL had a significantly higher number of CD41a+ platelets adherent to HMEC-1 cells compared to FL and HL in untreated chambers (Figure 6B). TNF-α as well as IL-6 increased the number of CD41a+ platelets in FL (*p* < 0.05) and HL (*p* < 0.001), but not in DLBCL (Figure 6B). In addition, the area of CD41a+ cells was increased in HL upon TNFα treatment (*p* < 0.01), indicating platelet aggregation (Figure 6C,D). However, the area of platelet aggregates was 2-fold increased in DLBCL in the presence of IL6 compared to the untreated control, which was not observed in FL and HL (*p* < 0.01, Figure 6C,D). Together, these results showed that TNF-α and IL6 increased platelet adhesion in FL and HL, while IL-6 increased platelet aggregation in DLBCL.

## 4. Discussion

Our study demonstrates that endothelial activation, inflammatory signaling, and dysregulated cytokine and NO pathways are closely linked to lymphoma pathophysiology and thrombotic risk. We found increased TF on circulating endothelial cells in patients with NHL, despite reduced plasma TF levels. Given that TF in lymphoma does not originate from malignant cells [34], these findings indicate endothelial cells as a key source of procoagulant activity. This is consistent with observations in venous thromboembolism, where activated circulating endothelial cells expressing CD142 correlate with TF expression and thrombotic burden [35]. The elevation of circulating endothelial progenitors (CEPs) in both Hodgkin lymphoma [36] and NHL [37,38], particularly in advanced or aggressive disease, further supports a central role for endothelial activation in lymphoma biology. Our data extends these reports by demonstrating that circulating endothelial cells are broadly elevated in lymphoma, suggesting a mechanistic link between endothelial activation, TF expression, and the well-recognized thrombotic risk. Clinically, these insights underscore the potential of endothelial-derived TF and CEPs as biomarkers for risk stratification and as targets for thromboprophylaxis. An intriguing finding of our study was the discordant pattern of endothelial activation markers in Hodgkin lymphoma, characterized by increased CD31+ CD146+ endothelial cells in the absence of concomitant upregulation of TF. This suggests that endothelial activation in HL may preferentially promote leukocyte adhesion and vascular permeability rather than a fully procoagulant phenotype. HL is distinguished by a highly cytokine-rich microenvironment dominated by non-malignant immune cells and sustained STAT3 and NFκB signaling, which strongly induce endothelial adhesion molecules such as CD146 but may differentially regulate coagulation pathways [18]. In contrast, DLBCL and FL exhibited a more classical prothrombotic endothelial phenotype, with concurrent upregulation of CD146 and TF, consistent with enhanced coagulation activation observed in these subtypes. Future work should determine whether endothelial TF is a driver of lymphoma-associated thrombosis or a secondary marker of vascular injury, and whether its modulation could alter patient outcomes. Thrombosis is a common complication in both malignant and non-malignant disorders, while the malignancy itself promotes a thrombophilic state through venous stasis, endothelial injury, and an imbalance of pro- and anti-thrombotic factors leading to hypercoagulability associated with poorer clinical outcomes and increased mortality [39].

In parallel, we observed that plasma uPA levels were elevated in lymphoma patients, in contrast to tPA. In the present study, reduced plasma tPA levels were already observed in DLBCL patients without clinically documented thrombosis, suggesting that impairment of fibrinolytic capacity may precede overt thrombus formation rather than represent a secondary consequence of thrombosis. In patients with established thrombosis, tPA levels may be influenced by endothelial activation, consumption within the thrombus, or compensatory release, which could account for the absence of statistically significant differences between DLBCL patients with and without thrombosis. This also aligns with prior evidence linking the uPA system to tumor aggressiveness and poor survival [40] and with reports of high uPA promoting progression and metastasis in cancers [41]. Importantly, these findings position uPA not only as a biomarker of lymphoma development and progression but also as a potential driver of the prothrombotic and invasive phenotype. We showed that uPA negatively correlated with the overall survival of patients with FL, indicating that heightened uPA activity may reflect aggressive tumor–microenvironment interactions rather than a compensatory fibrinolytic response. The significant increase in uPA levels in DLBCL patients with thrombosis likely reflects endothelial activation and tissue remodeling secondary to thrombus formation, rather than a sole initiating factor, although contributions to thrombus propagation and resolution cannot be excluded. Clinically, circulating uPA could aid in risk stratification and may represent a therapeutic target, though further studies are required to clarify whether its elevation reflects tumor burden or active disease promotion. Furthermore, these findings reinforce the idea that a reduction in tPA in DLBCL reflects an early prothrombotic milieu that may contribute to subsequent thrombus development.

We found that NFκB and p38 MAPK inflammatory signaling pathways were broadly activated in lymphoma MNCs, with preferential upregulation of STAT in DLBCL patients with thrombosis. The pronounced STAT3 activation observed in thrombotic DLBCL may therefore contribute indirectly to a prothrombotic phenotype by enhancing platelet–endothelial crosstalk, a hypothesis that requires confirmation in future functional studies. The p38 MAPK activation has been implicated in both DLBCL and FL and proposed as a therapeutic target [42], while its interaction with NFκB is essential for TNF-α-driven survival of lymphoma cells [43]. NFκB activation via phosphorylated p65 has been linked to a favorable prognosis in DLBCL [44], in contrast to STAT3 activation, which correlates with poor survival in treated patients [45]. These opposing associations highlight the complexity of pathway crosstalk. Notably, NFκB-induced IL-6 expression can sustain STAT3 activation in DLBCL, underscoring their cooperative role in lymphoma biology [20]. Activated NFκB signaling positively correlated with INR. These findings extend this evidence by demonstrating preferential activation of these pathways in thrombosis-associated DLBCL, suggesting a mechanistic link between inflammatory signaling, disease progression, and thrombotic risk. Targeting these signaling networks may therefore hold therapeutic promise while offering potential biomarkers for risk stratification. Our study allows us to propose a two-hit model of thrombosis in which all lymphoma subtypes are characterized with activated inflammatory NFκB and p38 MAPK signaling and increased endothelial activation, leading to increased thrombosis risk. Moreover, TNFα decreased trans-endothelial migration of lymphoma MNCs in an in vitro model regardless of thrombosis occurrence. Although NFκB and p38 MAPK activation are hallmarks of systemic inflammation, both pathways directly regulate thrombotic mechanisms, including endothelial tissue factor expression, platelet–endothelial interactions, and fibrinolytic imbalance, supporting their role as mediators of thrombo-inflammation rather than nonspecific inflammatory markers alone. Inflammatory signaling pathways, including NFκB, STAT3, and p38 MAPK, occupy a central position in the bidirectional interplay between immune cells and the endothelium. Activation of these pathways in circulating mononuclear cells can precede endothelial dysfunction by driving cytokine production, leukocyte adhesion, and tissue factor expression. At the same time, endothelial activation and barrier disruption further amplify inflammatory signaling in immune cells, establishing a self-reinforcing thrombo-inflammatory circuit. The elevated activation of NFκB, STAT3, and p38 MAPK observed in DLBCL patients with thrombosis is therefore consistent with both upstream immune-driven endothelial activation and downstream amplification following endothelial dysfunction.

Cytokine dysregulation further reinforces these pathogenic mechanisms. We found that trans-endothelial migration of MNCs was increased in HL and FL and further enhanced by thrombosis in DLBCL, but in both settings attenuated by TNF-α. Alongside this, TNF-α and IL-6 promoted platelet aggregation, linking inflammatory signaling to the prothrombotic phenotype of lymphoma. Elevated serum IL-6 and TNF-α have been consistently reported in HL and NHL [46,47], and higher levels correlate with greater tumor burden, aggressive disease, and inferior survival in FL and DLBCL [48,49]. Moreover, TNF-α expression in DLBCL tumor cells predicts poorer overall survival [50]. Genetic studies further support a role for TNF signaling, with the TNF-α promoter polymorphism G-308A and NFKB1 variants associated with increased NHL risk [51,52]. Taken together, our findings highlight TNF-α and IL-6 as central mediators at the intersection of inflammation, thrombosis, and lymphoma progression, with potential value as both prognostic biomarkers and therapeutic targets.

Finally, NO signaling is altered in lymphoma. IL-6 expression and cGMP-dependent NFκB activity in MNCs are modulated by NO, being enhanced at low and suppressed at high concentrations [53]. In DLBCL, we found that iNOS expression was elevated in MNCs, whereas plasma NO metabolites were increased in FL, but reduced in other forms of lymphoma. It is reported that NO increased trans-endothelial–MNC migration [54]. B-cell neoplasms express neuronal NOS and iNOS isoforms and nitrotyrosine, indicating active NO-driven processes within the tumor microenvironment [55]. Elevated exhaled NO and iNOS/eNOS expression in inflammatory cells further supports a role for NO in HL-associated inflammation [56]. NO positively correlated with fibrinogen, CRP and PLR in DLBCL as coagulation and inflammation biomarkers. NO bioavailability reflects the balance between protective endothelial NO production and inflammatory dysregulation. In FL, elevated plasma nitrite levels may indicate preserved or compensatory eNOS activity, consistent with the indolent nature of the disease and relatively intact endothelial function. In contrast, DLBCL and HL are characterized by intense systemic inflammation and oxidative stress, conditions known to reduce NO bioavailability through eNOS uncoupling and rapid NO scavenging. Thus, reduced NO levels in these aggressive lymphoma subtypes likely represent endothelial dysfunction rather than reduced NOS expression, contributing to a prothrombotic vascular phenotype. Functionally, iNOS activity can limit CAR T-cell efficacy, as pharmacologic inhibition improves responses, while high levels of iNOS+ CD14+ monocytes predict non-durable responses in large B-cell lymphoma [57]. Moreover, strong iNOS expression in Hodgkin and Reed–Sternberg cells correlated with shorter overall survival [58]. The obtained results suggest that elevated NO levels could function as a predictive biomarker of thrombotic risk, potentially reflecting increased oxidative and inflammatory signaling rather than protective endothelial NO production. The addition of nitrite levels provides unique biological information not captured by the clinical parameters of the ThroLy and Khorana scores, offering a more accurate and reliable method for risk stratification. Moreover, these findings suggest that dysregulated NO signaling contributes to lymphoma pathogenesis, influences inflammatory and immune interactions, and may represent a targetable mechanism to enhance therapeutic response.

Our study has several limitations. First, we were unable to fully correlate the experimental findings with comprehensive laboratory and clinical data due to the limited number of experiments performed. Second, trans-endothelial migration assays of MNCs were conducted using a single inflammatory cytokine, which may not fully capture the complexity of cytokine interactions in vivo. Third, although we assessed multiple signaling pathways, including NFκB/STAT3/p38, TNF-α/IL-6, and NO/NOS, the crosstalk between these pathways in patient samples was not fully explored, limiting mechanistic interpretation. Also, we collected the relatively small number of healthy controls, which may reduce the statistical power of comparisons with patient groups. Finally, our findings are based on ex vivo and in vitro assays, and their direct translation to clinical outcomes or therapeutic interventions requires further validation in larger patient cohorts and in vivo models.

## 5. Conclusions

As a central driver of coagulation, TF associates with circulating endothelial cells in lymphoma and promotes inflammation through NFκB and p38 MAPK, with STAT3 activation positively correlating with the clinical stage. TNF-α impaired mononuclear cell migration, sustaining a prothrombotic state by limiting thrombus resolution and endothelial repair. Inflammatory cytokines enhanced platelet aggregation, while reduced tPA with compensatory uPA disrupted fibrinolysis. Elevated endothelial cells and NO were not matched by tPA and eNOS, but activated monocytes produced iNOS and uPA in response to pro-inflammatory cytokines TNF-α and IL-6. Collectively, these findings show that chronic inflammation underlies lymphoma-associated thrombosis, where endothelial activation, cytokine imbalance, and monocyte-driven uPA/iNOS converge with NFκB, STAT3, and p38 MAPK pathways. These mechanisms highlight potential biomarkers and therapeutic targets, offering opportunities for improved risk stratification and tailored interventions in lymphoma patients.

## Figures and Tables

**Table 1:** Correlation between inflammatory signaling pathways and clinical parameters of patients with lymphoma.

Lymphoma		DLBCL			DLBCL + Thrombosis	FL	HL					
Parameters (P)		NLR	Khorana Score	Clinical Stage	MPV	ThroLy Score	HGB	MCHC	Albumin	Khorana Score		
STAT3 Spearman	r	−0.654	−0.666	−0.762	0.555		−0.58	−0.64	−0.59	−0.971		
	95% CI	−0.9–−0.07			0.12–0.81		−0.87–0.003	−0.89–−0.09	−0.87–−0.002			
	*p*	0.034	0.043	0.025	0.014		0.049	0.03	0.048	0.011		
STAT3 Pearson	P	aPTT	Fibrinogen						BA%			
	r	−0.63	−0.61				−0.628	−0.733	0.585	−0.957		
	r^2^	0.395	0.37				0.395	0.537	0.342	0.916		
	95% CI	−0.89–−0.046	−0.89–−0.013				−0.88–−0.08	−0.92–−0.27	0.016–0.87	−0.1–−0.65		
	*p*	0.038	0.047				0.029	0.007	0.046	0.003		
NFκB Spearman	P						WBC	NE%	ANC	PTs	PT%	INR
	r					0.7	0.692	0.608	0.72	0.62	−0.72	0.748
	95% CI						0.178–0.91	0.034–0.88	0.231–0.92	0.052–0.88	−0.92–−0.19	0.288–0.93
	*p*					0.022	0.016	0.04	0.011	0.035	0.016	0.007
N^o^ pts		11	10/11	9	19	10	12	12	12	6/12	11	12

Absolute neutrophil count (ANC), nuclear factor kappa B (NFκB), signal transducer and activator of transcription (STAT), follicular lymphoma (FL), Hodgkin lymphoma (HL), diffuse large B-cell lymphoma (DLBCL), neutrophil-to-lymphocyte ratio (NLR), mean platelet volume (MPV), mean corpuscular hemoglobin concentration (MCHC), white blood cells (WBCs), international normalized ratio (INR), percentage of neutrophils (NE%), hemoglobin (HGB), percentage of basophils (BA%), prothrombin time (PTs), prothrombin time percentage (PT%), activated partial thromboplastin time (aPTT). Correlation coefficients (r), 95% confidence intervals (CIs), and *p*-values are shown.

## Data Availability

The data presented in this study are available on request from the corresponding author because they are not publicly available due to privacy and ethical restrictions.

## Supplementary Materials

The following supporting information can be downloaded at https://www.mdpi.com/article/10.3390/cells15080667/s1, Figure S1: Levels of endothelial progenitor cells in lymphomas. Using flow cytometry, we determined levels of CD31, CD142 and CD144 markers of endothelial cells in diffuse large B-cell lymphoma (DLBCL), follicular lymphoma (FL) and Hodgkin lymphoma (HL) in comparison to healthy volunteers (control). These gating images for CD31, CD142 and CD144 markers distribution, determined by flow cytometry, correspond to Figure 1. Figure S2: Receiver operating characteristic (ROC) curve showing the diagnostic performance of (**A**) pSTAT3/STAT3 ratio, (**B**) nitrite levels, (**C**) combined ThroLy score and nitrite model, (**D**) combined Khorana score and nitrite model, (**E**) ThroLy score, and (**F**) Khorana score in distinguishing patients with lymphoma from healthy controls. Figure S3: Single-stain controls for endothelial HMEC-1 cells, mononuclear cells, and platelets.
