# Supplementary material for: Inflammatory Signaling and Endothelial Activation Drive Thrombosis in Hodgkin and Non-Hodgkin Lymphoma
Source: Cells. 2026 Apr 9;15(8):667. doi: 10.3390/cells15080667 (PMC13114949; doi:10.3390/cells15080667)
Supplement: Supplementary file 1 [file cells-15-00667-s001.zip › cells-4146517-supplementary.pdf]

Supplemental Figure S1

A **B** **C** **D**

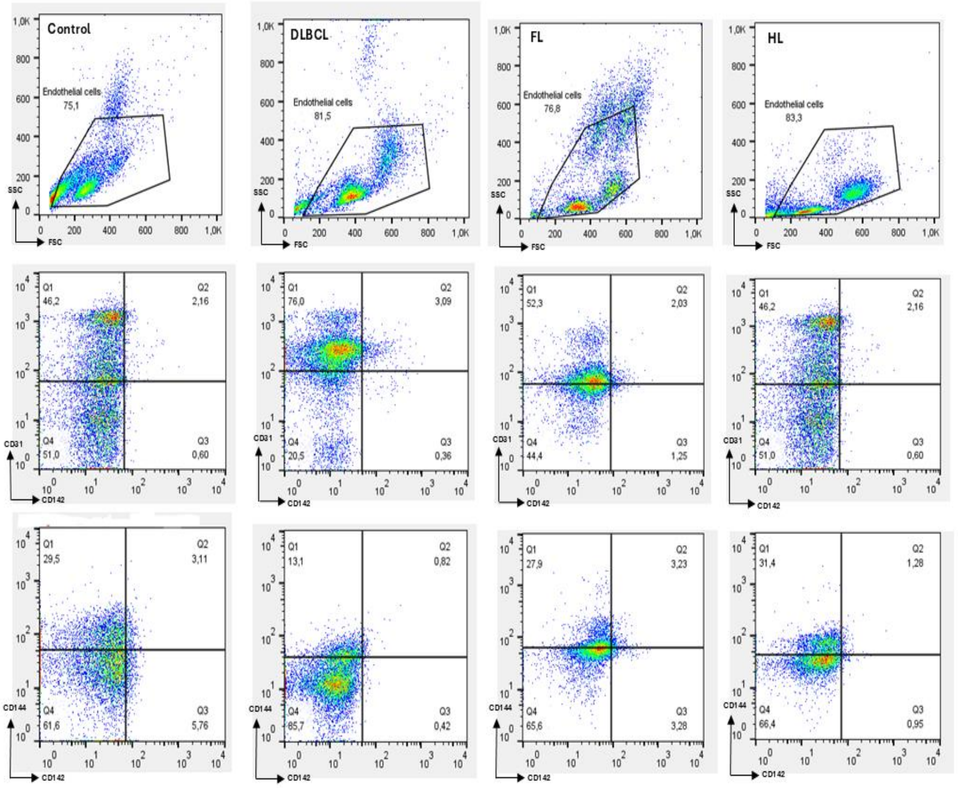

Supplemental figure S1. Tissue factor expression in circulating endothelial cells in lymphoma patients. Using flow cytometry, we determined levels of endothelial CD31 and CD144 markers and tissue factor (CD142) in A) healthy volunteers (control) B) diffuse large B-cell lymphoma (DLBCL), C) follicular lymphoma (FL) and D) Hodgkin lymphoma (HL). These gating images for CD31, CD144 and CD142 markers distribution, determined by flow cytometry, correspond to Figure 2.

Supplemental Figure S2

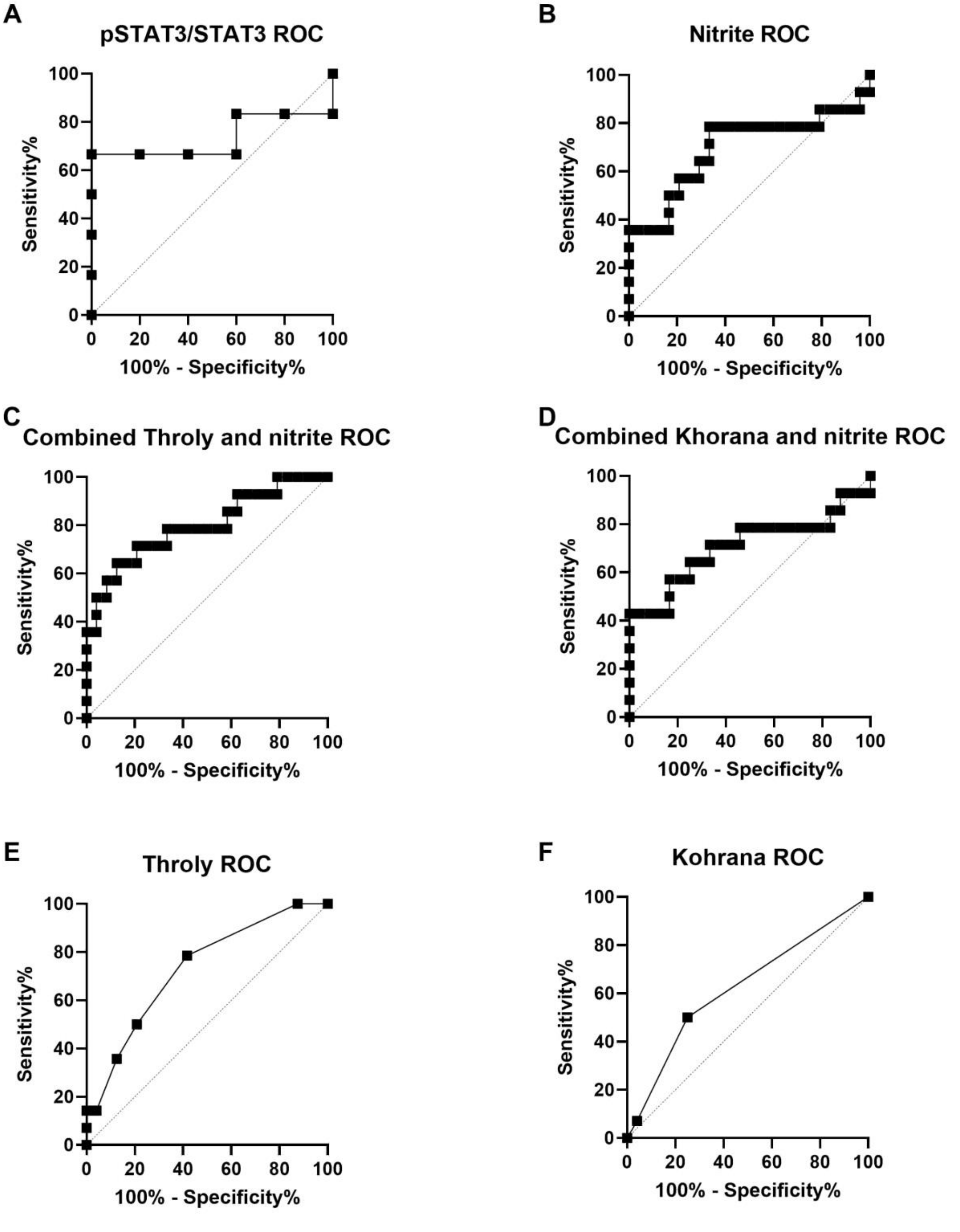

Supplemental figure S2. Receiver operating characteristic (ROC) curve showing the diagnostic performance of A) pSTAT3/STAT3 ratio, B) nitrite levels, C) combined ThroLy score and nitrite model, D) combined Khorana score and nitrite model, E) ThroLy score, and F) Khorana score in distinguishing patients with lymphoma from healthy controls.

Supplemental Figure S3

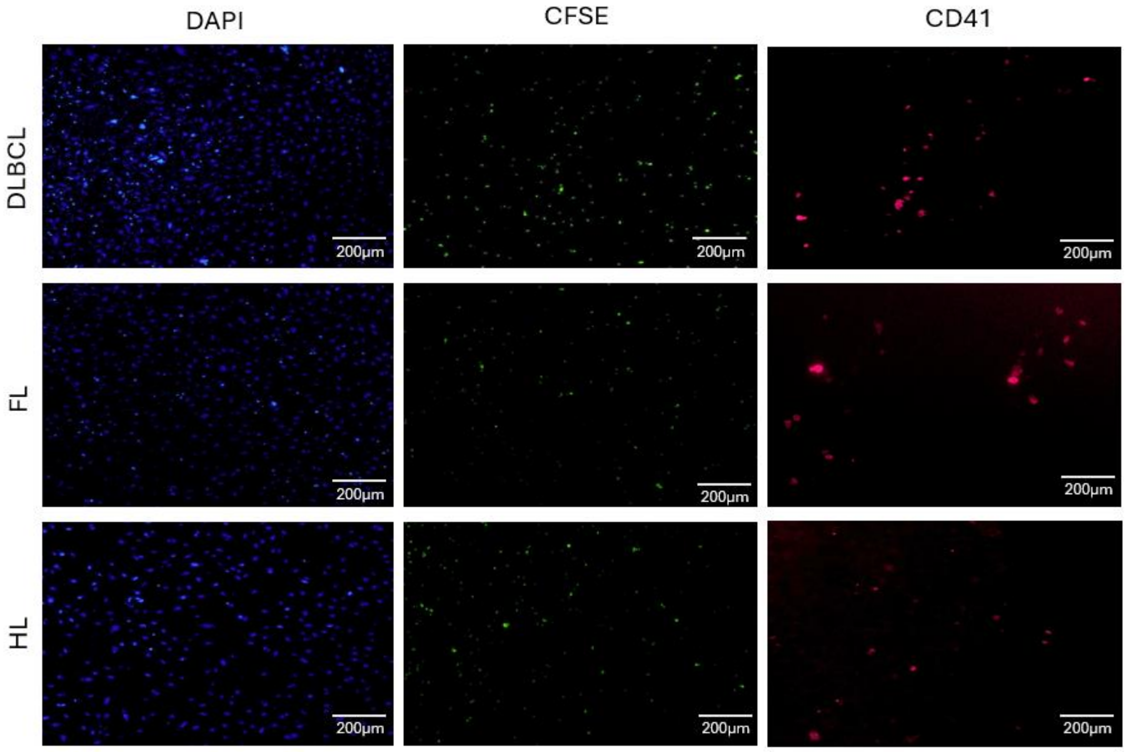

Supplemental figure S3. Single-stain controls for endothelial HMEC-1 cells, mononuclear cells, and platelets of lymphoma patients. Representative fluorescence microscopy images showing nuclear staining with DAPI (blue), CFSE labeling (green), and CD41 expression (red) in samples derived from diffuse large B-cell lymphoma (DLBCL), follicular lymphoma (FL), and Hodgkin lymphoma (HL). DAPI highlights HMEC-1 nuclei, MNCs labeled with CFSE, and platelet aggregates stained with CD41 antibody.
